# Durability of Externally Bonded Fiber-Reinforced Polymer Composites in Concrete Structures: A Critical Review

**DOI:** 10.3390/polym13050765

**Published:** 2021-02-28

**Authors:** Jovan Tatar, Sandra Milev

**Affiliations:** Department of Civil & Environmental Engineering, Center for Composite Materials, University of Delaware, Newark, DE 19716, USA; milev@udel.edu

**Keywords:** FRP, composites, durability, degradation, civil infrastructure, concrete, repair, retrofit, strengthening

## Abstract

Externally bonded fiber-reinforced polymer composites have been in use in civil infrastructure for decades, but their long-term performance is still difficult to predict due to many knowledge gaps in the understanding of degradation mechanisms. This paper summarizes critical durability issues associated with the application of fiber-reinforced polymer (FRP) composites for rehabilitation of concrete structures. A variety of factors that affect the longevity of FRP composites are discussed: installation, quality control, material selection, and environmental conditions. Critical review of design approaches currently used in various international design guidelines is presented to identify potential opportunities for refinement of design guidance with respect to durability. Interdisciplinary approaches that combine materials science and structural engineering are recognized as having potential to develop composites with improved durability.

## 1. Introduction

Externally bonded (EB) fiber-reinforced polymer (FRP) composites are one of the most economical technologies showing promise to recover deteriorated concrete structures as well as improve the resilience of critical infrastructure across the world. EB FRP can be used to upgrade columns, beams, and walls in a variety of concrete structures ranging from residential and commercial buildings to critical infrastructure (e.g., roads, bridges, tunnels, and marine structures, etc.) [1]. FRP composites consist of fibers that are embedded in a polymer matrix (resin). The fibers provide strength and stiffness to the material, while the resin ensures fiber alignment, transfers stress between the fibers, and provides environmental protection for the fibers. The composites are externally bonded to a structural member’s surface with a resin similar to that used to form the composite matrix.

**Figure 1:**
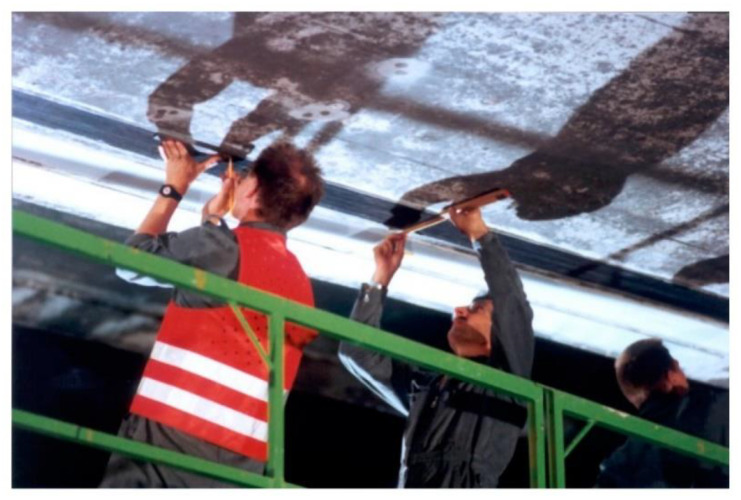
Application of externally bonded (EB) fiber-reinforced polymer (FRP) strip on Ibach bridge near Lucerne in Switzerland in 1991, what is believed to be the first application of EB FRP in the world (reproduced from [4]).

Even though composites have been in use in civil engineering for close to 30 years [2,3], EB FRP composite strengthening systems are still a relatively unknown practice in the civil engineering community at large. Potential limiting factors to their widespread adoption are the lack of comprehensive design standards and long-term test data warranting the durability of these systems. Since the very early EB FRP applications, concerns have been raised about the long-term durability of these materials, especially when faced with a combined effect of sustained load, fatigue, and environmental factors—typical for outdoor applications (Figure 1).

Paradoxically, exceptional durability of composites is often cited as one of the main reasons for the use of FRP over other materials in aerospace, automotive, and marine industries. Although composites have been successfully used in these industries without experiencing any significant durability problems, it should be noted that these “parent” composite systems quite significantly differ from those used in infrastructure applications nowadays. Quality of constituent materials, processing and curing conditions, and environmental loadings are often significantly different. For instance, to drive down the cost and constrained by the application-specific requirements, civil engineers resort to the wet-layup application process (over resin transfer molding and autoclave molding) and resins such as ambient-cured epoxies (over high-temperature cured epoxies) [5,6]. These choices make EB FRP composites cheaper but inferior in performance over those used in the aforementioned industries.

Furthermore, materials used in the aerospace industry have to pass conservative specification and qualification testing requirements to yield extraordinarily high levels of safety (i.e., low probability of failure) [7,8]. The qualification testing protocols involve extensive durability testing (e.g., accelerated conditioning) with an intention to prescribe material design properties with a low probability of exceedance, rather than accurately evaluating the composites’ deterioration over time. In civil engineering applications, where decisions are primarily cost-driven, meeting such extraordinarily high requirements of materials qualification would significantly increase EB FRP costs and make them uncompetitive in comparison to traditional construction materials such as concrete and steel. Similarly, using excessively high material design safety factors drives up the overall costs of EB FRP strengthening systems and makes them a less viable solution for structural upgrading. There is, thus, a desire to accurately characterize composites’ degradation to ensure less conservative designs and make EB FRP more competitive in a cost-driven decision-making process. 

Across the world, there is a rising need to implement EB FRP in harsh environmental conditions in concrete structures to prolong their service life and ensure hazard resilience. Some example applications of EB FRP in harsh environments include concrete bridges and dams, water-treatment facilities, and nuclear reactors, where EB FRP composites must endure combined effects of multiple environmental stressors and structural loadings while maintaining sufficiently long service life. Civil engineers designing such structural upgrading systems are often faced with a lack of guidance regarding the proper treatment of durability concerns. The intent of this paper is to provide an overview of the most common deterioration mechanisms in the EB FRP systems and a critical assessment of the existing design guidance worldwide for EB FRP materials used in harsh environmental conditions. The reader shall be aware that, even though care is taken to provide the most up to date information, the research in this area is still ongoing while new materials, durability design guidelines, and test methods are under development.

## 2. Current State of Practice: EB FRP Installation

Installation of EB FRP is a relatively simple process, but even relatively small deviations from the prescribed procedures can cause defects in the bonded systems that can lead to poor performance of the EB FRP in the long term. Usually, the most critical component of the system governing its effectiveness is bond quality [9] between EB FRP and concrete which is a function of installation procedure, environmental conditions, and adhesive and substrate materials quality.

### 2.1. Bond-critical vs. Contact-Critical

**Figure 2:**
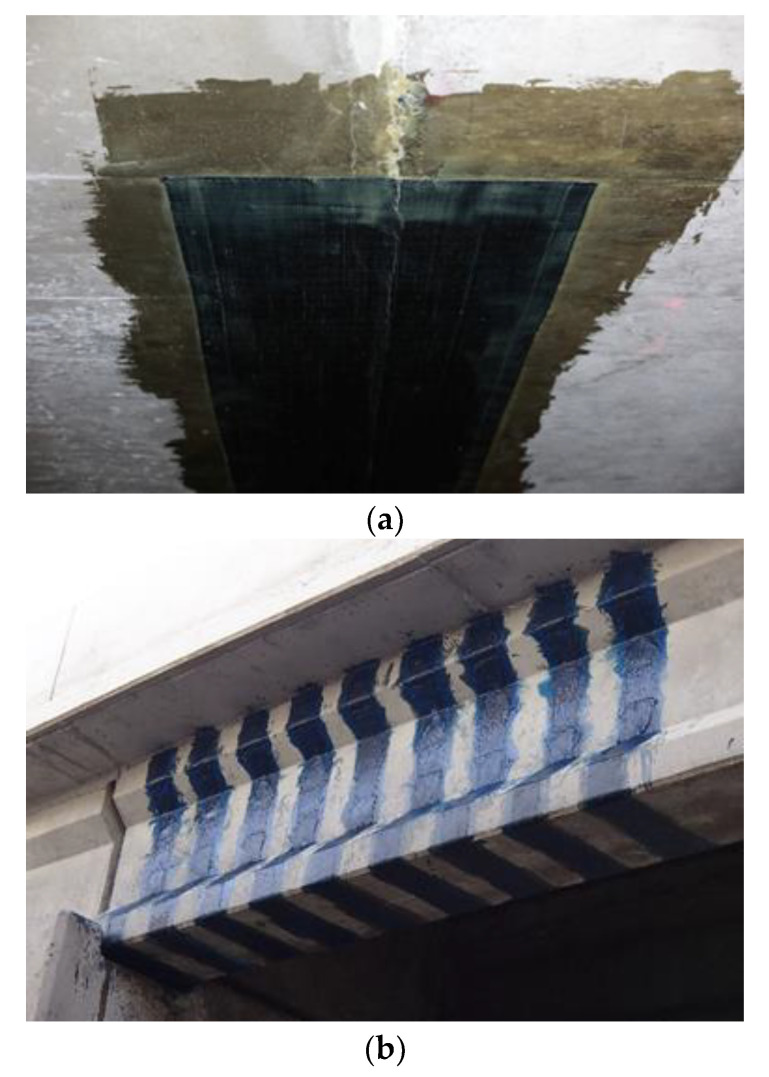
Examples of bond-critical applications of EB FRP: (**a**) flexural strengthening of a concrete slab in a parking garage and (**b**) shear strengthening on the Sunshine Skyway bridge in Tampa, FL, USA.

**Figure 3:**
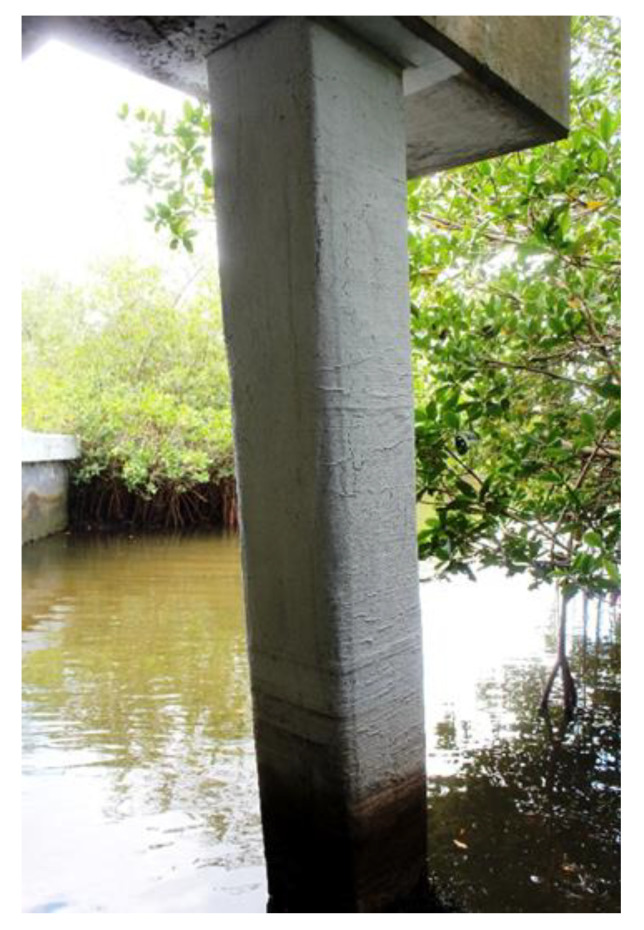
Example of contact-critical application of EB FRP: FRP-confined column in a Bridge in Florida, USA (note: FRP wrap is painted).

EBFRP applications require strong adhesive bonding between EB FRP and the concrete substrate for effective stress transfer between the adherents; examples of such applications include flexural [10] and shear strengthening [11] (Figure 2). Contact-critical applications warrant an intimate contact between EB FRP and concrete while the strength of the bond between EB FRP and concrete is not as crucial. The most typical contact-critical application is FRP confinement in columns (Figure 3). With respect to environmental durability—in bond-critical applications—both the durability of composite and composite-concrete adhesive bonds play an essential role [12,13]. In contact-critical applications durability of the composite is a more important variable than composite/concrete bond due to the passive confinement being provided by the composite [14].

### 2.2. Surface Preparation and Installation

**Figure 4:**
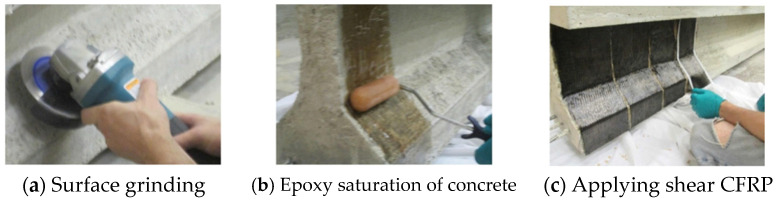
(**a**–**c**) EB FRP installation (reprinted from [24]).

Whether the EB FRP application is bond-critical or contact-critical will affect the importance of proper concrete surface preparation. Even though substrate preparation and quality of adhesive bonding are more important in bond-critical applications, recent evidence shows that defects at the EB FRP/concrete interface can significantly deteriorate the strength of EB FRP-confined columns [15]. As bond-critical applications rely on strong adhesive bonding between FRP and concrete, it is essential to ensure adequate conditions in the concrete substrate are being met to allow for strong adhesion to occur [16,17]. Although multiple types of EB FRP systems have been developed, the most utilized systems are wet-layup EB FRP systems (Figure 4) due to the ease of application under in-situ conditions, and their ability to conform to various geometric shapes and configurations (in both bond-critical and contact-critical applications). Further discussion will mainly focus on wet-layup applications.

The EB FRP installation process starts with concrete surface preparation (Figure 4a). Depending on the quality of the existing concrete substrate, the general recommendation is to (1) remove the defective, damaged or deteriorated concrete, (2) repair defective steel reinforcement, and (3) restore the concrete section [18]. The concrete surface is then roughened (via sandblasting, grinding, needle scaling, etc.) to achieve the desired level of roughness and expose the aggregate. The United States (U.S.) construction specifications recommend a minimum surface roughness corresponding to the International Concrete Repair Institute (ICRI) Concrete Surface Profile chip No.3 (ACI 440.2R) [19]. Example documents providing detailed guidance on concrete surface preparation are ACI 546R and ICRI No. 310.2R [20,21]. It is also recommended that any sharp corners and inside and outside edges be rounded or chamfered to an adequate radius of curvature to minimize stress concentrations in EB FRP and prevent the formation of air pockets between the EB FRP and concrete during installation [18,19]. Any surface imperfections (bug holes, large protruding aggregate grains, surface indentations, etc.) shall be smoothed and/or filled with paste epoxy (epoxy adhesive with mineral fillers also known as “putty”) to ensure even surface. The final stage in surface preparation consists of surface cleaning to remove any dirt, laitance, debris, oil, etc., and is usually performed by pressure washing and blowing by compressed air.

Following concrete surface preparation, the epoxy primer is applied to the concrete surface (Figure 4b). The primer can be the same epoxy used to saturate the dry fiber fabric, or it can be specifically formulated to promote adhesion between EB FRP and the concrete substrate. Presence of water on the concrete surface can significantly affect the adhesion between the primer and concrete [22]. Thus, NCHRP 514 recommends that concrete surface moisture at primer application should be below 0.05% (as measured by surface moisture meter). Although water-resistant primers exist on the market, limited experimental evidence suggests that they are not entirely successful at mitigating adhesion loss due to moisture present in the substrate [22]. The existing installation guidelines furthermore suggest that if a concrete surface is subjected to moisture vapor transmission, EB FRP should not be installed as vapor transmission can cause blistering along the EB FRP-concrete bondline [19,23]. 

Before application to a primed concrete surface, dry fiber fabric is impregnated with a resin either by hand (using a special saturating roller) or via resin impregnation machine. Impregnated EB FRP fabric can be applied directly to the primed surface (Figure 4c), or it can be preceded by application of a putty layer to even out the surface and remove any geometric imperfections. Special care should be taken to achieve proper fiber orientation while ensuring FRP is spread without creases and entrapped air bubbles. 

After installation, EB FRP is allowed to cure according to the manufacturer’s specification. Given that the resin cure is dependent on the ambient temperature, the proper cure may require several days. In certain situations, it is prudent to apply an appropriate coating to cured EB FRP surface for aesthetic purposes and as protection from ultra-violet (UV) exposure and other environmental factors. While the ability of the protective coating to preserve the durability of EB FRP is often assumed, experimental evidence proving their effectiveness is rather scarce.

### 2.3. Quality Control

**Figure 5:**
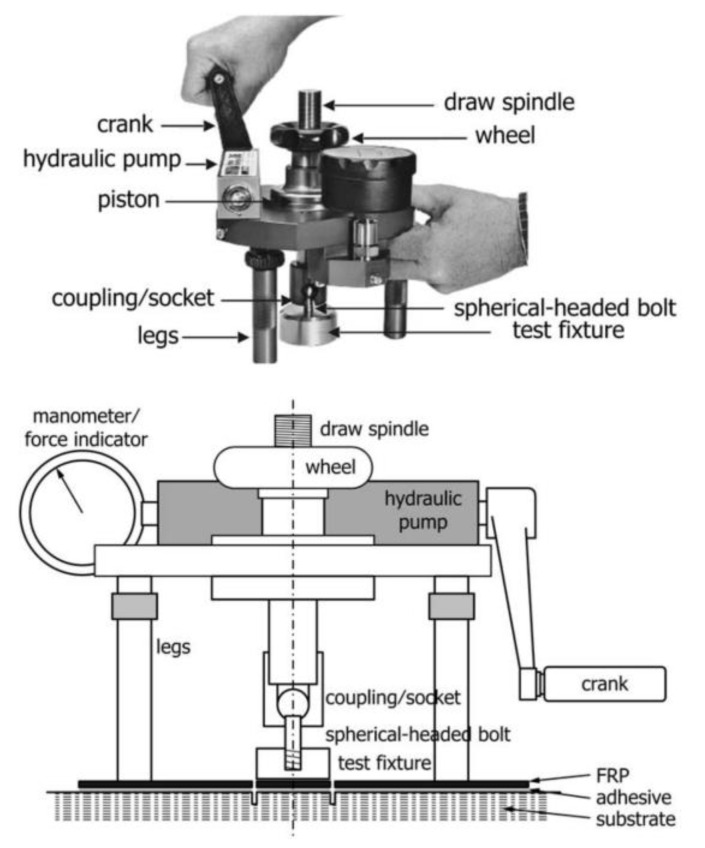
Pull-off test setup (reproduced, with permission from [31]).

**Figure 6:**
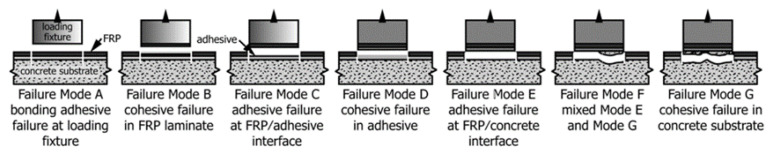
Pull-off test possible failure modes (reproduced, with permission from [31]).

Following installation and initial cure, the current state of practice in the U.S. is to perform a visual inspection for any signs of debonding or incomplete resin cure, which is followed by a pull-off test per ASTM D7522 (Figure 5). Common practice is to accept tests with pull-off strength exceeding 1.4 MPa (200 psi) with a failure within the concrete substrate (Failure Mode G per Figure 6) [25]. ACI 440.2R further recommends that test results below 1.4 MPa (200 psi) or failure mode other than “G” be submitted to a licensed design professional for evaluation and acceptance. The quality control guidance usually recognizes that proper bonding may not be as important in contact-critical applications. However, the authors of this article encourage enforcement of the same evaluation criteria in contact-critical applications for the overall promotion of quality (and consequent longevity) in such EB FRP applications.

Conducting pull-off tests is expensive and time-consuming while also not being entirely non-destructive. Therefore, there is a need to develop rapid non-destructive test methods to facilitate evaluation of the adhesive bond. In addition, concerns have been raised about stress concentrations introduced by the tested locations [26]. Accordingly, these tests should be performed in locations where low stresses are to be expected. The tested locations are usually repaired by installing an EB FRP patch across the test site to ensure continuity of the composite.

In general, the lack of standardized techniques and procedures hinders development of inspection protocols and ability to quantify quality of the FRP installation. Possibility of using thermography [27], acoustic methods [28], and recently smart sensors (electric sensors, piezoelectric sensors [29], fiber optic sensors [30]) to measure displacements, strains, bond quality, and advancement of cure reactions has been also investigated by some researchers. Despite the fact that significant research effort has been undertaken in developing non-destructive methods using “smart” materials, more research is needed before this innovative approach is adopted by practitioners.

## 3. Materials Selection, Environmental Exposure, and Load Conditions

**Figure 7:**
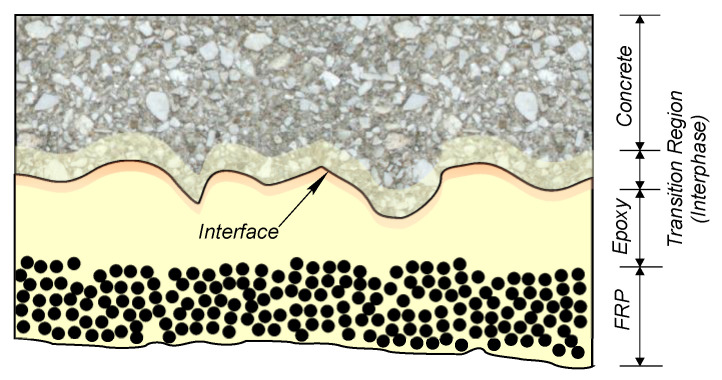
Schematic representation of a cross-section of FRP externally bonded to concrete.

EB FRP is a complex multilayer system consisting of three constituent materials and their corresponding interfaces. As schematically shown in Figure 7, the bonded joints typically consist of concrete, epoxy, and the FRP composite. Due to the concrete substrate porosity, a relatively prominent interphase (“transition region”) is formed between the adhesive and concrete substrate. Even though much smaller in size, interphase between fibers and polymer matrix plays a significant role in the overall performance and long-term durability of the FRP composite [32,33]. Given its multiscale nature and possible deterioration mechanisms of the individual constituent materials (concrete substrate, epoxy, and the FRP composite), the possible deterioration mechanisms in an FRP-concrete bonded system can be highly complex. While the overall performance of EB FRP is likely to be affected by deterioration in either of the system components (concrete, epoxy, FRP composite), transition region that forms along the bondline between epoxy and the concrete substrate, as well as the fiber-epoxy interphases, can govern the durability performance of the bonded system [34,35]. Evaluation of durability of FRP-concrete bonded joint is not as simple as studying the durability of each of the system components (concrete, epoxy, FRP) separately. The problem, instead, requires an evaluation of durability at both the component and system levels given the complexity of the system. 

During their service, EB FRP systems are exposed to a variety of environmental conditions, many acting simultaneously. High humidity, elevated temperature, freeze-thaw cycles, UV radiation, and de-icing agents can affect the performance of an EB FRP strengthening system [36,37,38,39]. So far, the effect of these exposure conditions has been mostly studied in laboratory environment using accelerated testing. Some of the studies conducted on FRP composites aged in natural environment report contradictory results regarding the correlation between natural exposure and laboratory conditioning. Frigione et al. [40] reported that level of degradation was higher in artificially conditioned samples. Tatar and Hamilton [41] concluded that laboratory durability data provide pessimistic estimate of composites’ durability compared to field exposure data for short service life. Other researchers found that FRP degrades more under outdoor natural exposure compared to that in accelerated aging tests [42,43,44]. To be able to validate artificial aging tests to precisely predict service life, more data on aging of composites in natural conditions is necessary. In recent years, more data from field studies have become available [45,46,47,48].

Loading conditions (cyclic-loading and fatigue, sustained loading, and impact) is another important factor affecting long-term performance of FRP, since presence of load accelerates its degradation and shortens the service-life of the repaired structures [49]. Both fatigue and sustained loading magnify the effect of environmental actions [50,51,52]. For FRP applied to bridges or railroads, fatigue behavior is critical for their safety, since these structures are subjected to cyclic vehicle loading [50]. Type of the fiber is one of the parameters affecting fatigue performance, but matrix composition is much more important [53,54]. The expected decrease of tensile strength of GFRP composites is about 10% per decade, while CFRP and AFRP composites exhibit tensile strength degradation between 5 and 8% per decade. Moreover, a steeper decrease of stress with increasing number of cycles is observed in composites with low-modulus fibers, compared to ones with high modulus fibers. Testing of different types of resin: epoxy, polyester, phenolic, shows that epoxy resin has superior performance over other types of resin. Toughness of the epoxy has an important role in the fatigue performance of the composite—tougher matrices have poorer fatigue performance [54,55]. Design specifications use different approaches to control fatigue performance—ACI 440.2R and TR55 limit stress level in the FRP, CNR specifies reduction factor to be applied to debonding stress limits under static loading, and *fib* addresses this issue indirectly—through reducing stresses in the internal steel reinforcement [56]. Level of constant dead load, typical for civil engineering structures, should also be considered when it comes to long-term performance of FRP, due to stress-relaxation and creep. It should be pointed out that in FRP strengthened/retrofitted structures, FRP typically does not carry sustained load. Rheological properties of the FRP-strengthened system are dominated by the resin matrix. High level of sustained loading, undercured resin, and higher service temperature increase creep deformation which can lead to excessive deformation at the level of structural component [57,58]. FRP reinforced structures are susceptible to impact damage during their service life (collision with vehicles and flying objects). Impact may cause damage to (1) the fiber, which will significantly affect its capacity to carry loads; (2) the matrix, affecting its ability to transfer stresses to the fibers (it was measured that FRP coupons with damaged epoxy retain 80% of the initial strength); and (3) the substrate, when penetration through the composite occurs leading to the reduction of local mechanical properties [59,60].

### 3.1. Resins and Adhesives

Commonly utilized resins and adhesives in infrastructure applications are thermosetting polymers such as polyesters, vinyl esters, and epoxies. Although polyester and vinyl ester resins can be formulated to exhibit good mechanical properties, they can display excessive shrinkage during curing and are often susceptible to accelerated deterioration under moisture [61]. Additionally, it has been noted that these resins can also exhibit poor resistance to creep under sustained loading [61]. 

Modern wet-layup EB FRP systems almost exclusively utilize ambient-cured epoxy as a composite matrix as well as an adhesive between EB FRP and concrete. When properly formulated, ambient-cured epoxy exhibits superior mechanical properties and better chemical resistance than other types of resins. Epoxy resins can have a range of viscosities and can cure under ambient conditions with minimal shrinkage. The adhesive also exhibits good wetting ability and adhesion to a variety of engineering materials, including concrete. 

**Figure 8:**
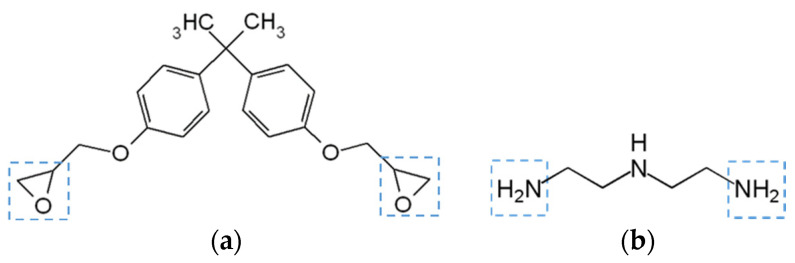
Epoxy adhesive precursors: (**a**) Bisphenol A diglycidyl ether (DGEBA) (epoxide groups marked with a square) and (**b**) example amine-based hardener–Diethylenetriamine (DETA) (amine groups marked with a square).

Epoxy is a two-component adhesive consisting of two precursors: (1) an epoxide group-containing polymer (or monomer) and (2) a curing agent or hardener. The backbone of the resin is usually a Bisphenol A diglycidyl ether (DGEBA) molecule which hosts epoxide groups (Figure 8a). Epoxide groups react with a hardener, commonly an amine-containing species (Figure 8b), which results in the onset of curing reaction where epoxide rings open and react with active functional groups of the hardener to form permanent covalent bonds, also known as crosslinks. The degree of cure of epoxy is often expressed in terms of conversion which represents the percentage of reacted epoxide functional groups. The density of the crosslinked network affects the adhesive’s mechanical properties and the temperature defining its transition from a glassy to a rubbery state (also known as glass transition temperature, or *T*_g_). Besides the monomer and hardener, epoxy adhesives often contain additives that can modify adhesive’s properties. Common types of additives in epoxy are accelerators (or curing promoters), coupling agents, antioxidants, and toughening agents.

**Figure 9:**
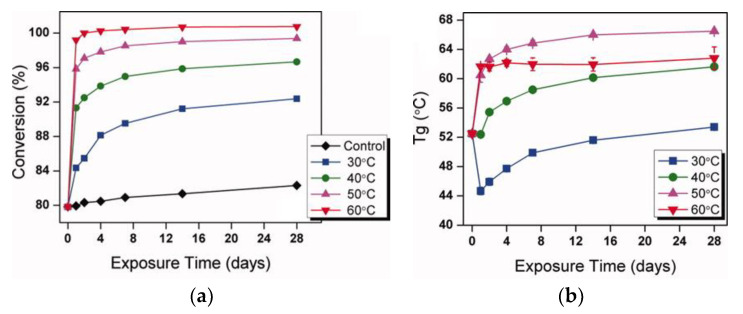
Change in the conversion (**a**) and *T*_g_ (**b**) of Epon 826/Jeffamine D-230 epoxy system over 28 days under standard laboratory conditions (“Control”) and water immersion at elevated temperatures (30, 40, 50, and 60 °C) (reprinted from [83]).

Durability properties of epoxy adhesives are not only affected by their service environment but also by the processing and curing conditions characteristic for in situ wet-layup applications [62,63,64]. Since epoxy adhesives are cured under ambient conditions, the properties of the adhesive are impacted by the environment in which it cures. For example, experimental evidence shows that epoxy adhesives cured under standard lab conditions often achieve less than 85% cure [65,66], while increasing the curing temperature (even under hygrothermal conditions) can significantly accelerate the conversion (Figure 9). The consequence of “slow” cure in ambient conditions is that long curing times may be necessary (especially in colder climates) [67,68] for the resin to achieve sufficient mechanical properties. It is, thus, strongly advisable that epoxy not be applied in ambient and concrete surface temperatures below 10 °C [18] to facilitate proper curing of the resin. Moreover, given that curing reaction is a temperature-dependent and diffusion-limited reaction, the full cure is usually never reached under ambient conditions, which results in adhesives whose *T*_g_ remains relatively low during the service life of EB FRP (usually between 55 and 75 °C) [66]. Given that concrete surface temperatures during summer months can be in the vicinity of 60 °C [69] or higher in many parts of the world, there is, thus, a possibility of service temperature exceeding the epoxy *T*_g_. This can lead to loss of resin’s mechanical properties, ultimately compromising the integrity of EB FRP/concrete adhesive bonding and stress transfer between the fibers and matrix.

Besides affecting epoxy’s mechanical and thermal properties, the incomplete cure may also render epoxy more vulnerable to certain deterioration mechanisms. Unreacted polar sites can “attract” water molecules into the cross-linked epoxy network that results in an onset of plasticization, which leads to a reduction in elastic modulus (by up to 50%), reduction in strength, and significant depression of *T*_g_ (as shown in Figure 9). Depending on the service temperature, plasticization (depressing *T*_g_) and post-cure (improving crosslinking density and increasing *T*_g_) are two competing mechanisms [66]. As can be seen in Figure 9, depending on the conditioning temperature, hygrothermal conditioning can result in either the depression or increase of *T*_g_ in the same resin. Further complicating the complexity of the problem, the effects of plasticization can be partially or fully recoverable [70,71,72].

In addition to their susceptibility to degradation under moisture, epoxy resins were also found to be sensitive to UV exposure, which leads to oxidation of the ether and nitrogen groups [73,74]. Oxidation is often accompanied by characteristic yellowing of the transparent resin as well as surface scaling and microcracking. UV exposure combined with hygrothermal conditioning was found to lead to hydrolysis [75,76]. Deterioration of adhesive and matrix resin in the FRP-reinforced structures due to long-term chloride exposure is also a concern, as it decreases the elastic modulus, tensile strength, and ultimate strain [77]. According to some studies, reduction of elastic modulus and tensile strength is larger in distilled water compared to saltwater [78]. However, the deterioration mechanism is still not clear and needs further research [79]. The effect of alkaline and salt solutions on adhesive durability was a subject of an extensive review by Yang et al. [80] Usually, exposure to deicing salts is accompanied with freeze-thaw cycles, which are major consideration when it comes to FRP composite and bond performance due to differential thermal expansion. In a study by Al-Mahmoud, epoxy resin was analyzed under SEM to better understand degradation of the bond between the FRP and concrete during freeze-thaw cycles. SEM images did not display any differences between the control sample and the samples exposed to freeze-thaw cycles. However, it has been reported in other studies that mechanical properties (tensile strength, ultimate strain, shear strength) of the resin after exposure to freeze-thaw cycles can reduce by 28%, 30%, and 60%, respectively [81]. When FRP composites are used in wastewater treatment plants, pipelines, or storage plants, resin matrices are exposed to acids. The durability of a resin in this case depends on the its chemical composition—vinyl ester resins show better resistance to acids than epoxy resins [82]. All of these deterioration mechanisms can significantly compromise epoxy’s strength, modulus of elasticity, fracture toughness, or adhesion properties.

### 3.2. Fibers and Composites

**Figure 10:**
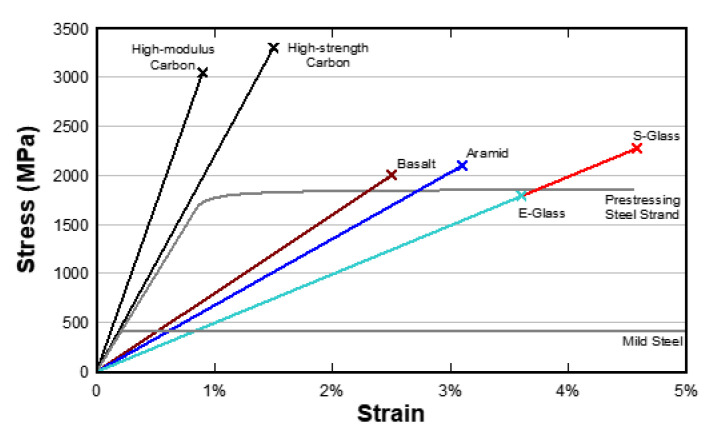
Comparison of different FRP composites to typical grades of steel used in concrete structures.

Comparison of stress-strain behavior for different types of FRP materials and common grades of steel used in construction is shown in Figure 10. Most commonly used fibers in EB FRP wet-layup applications are glass (E-glass and S-glass grades) and carbon fibers. Aramid fibers, while offering slightly better mechanical properties than glass, are significantly more expensive while presenting significant durability concerns [84,85]. Basalt fibers are an attractive alternative to glass fibers due to their improved mechanical properties and lower carbon footprint [86]. However, investigations are underway to elucidate their performance retention under typical infrastructural environmental exposures. Design guidelines also do not provide significant guidance on the design of strengthening systems with externally bonded basalt fiber reinforced polymers (EB BFRP). 

Glass fibers are economical and thus the most attractive for civil engineering applications. Their durability properties were thoroughly researched due to the desire in the construction industry to replace corrosion-susceptible mild steel reinforcement with non-corrosive glass fiber reinforced polymer (GFRP) bars [87]. Typically used grades of glass fibers (E-Glass and S-Glass) in civil engineering applications have relatively poor resistance to moisture, alkali, and acids [88,89,90]. Moisture uptake by the matrix in GFRP composites was found to cause plasticization of the matrix and subsequent reduction in elastic modulus. Moisture ingress can also induce cracking of the fiber-matrix interface through matrix swelling, osmotic pressures, and weakening of matrix-fiber chemical bonds [88]. However, by far the most detrimental environment for GFRP composites in concrete structures is high alkalinity (>13.5) typical for concrete leachate solution [91] which can lead to multiple deterioration mechanisms—primarily breakage of silica (SiO_2_) chains due to their reaction with hydroxide ions (OH^−^) as well as hydrolysis of the glass network by OH^−^ [92]. It is also well-established that typical glass fibers and their composites are susceptible to stress corrosion cracking [93,94]. In the design of EB GFRP, the stress level under service loads is usually limited to avoid creep-rupture failure of the composite (e.g., ACI 440.2R).

Corrosion-resistant (ECR-Glass) and alkali-resistant (AR-Glass) grades of glass fibers can alleviate some of the observed durability issues. While ECR- and AR-Glass offer better durability characteristics than E- and S-Glass, these fibers are still characterized by a low modulus of elasticity (in comparison to carbon) which limits their applicability in situations where EB FRP is used to address the serviceability (e.g., deflections and stiffness) concerns. When compared to CFRP, GFRP composites are more susceptible to environmental degradation especially when immersed in solutions and they are not adequate for application in aggressive environment [79]. When it comes to the effect of freeze-thaw, a small decrease in tensile strength was measured in GFRP coupons-only 3% by Sheikh et al. [95]. In the same study, tensile strength decrease of CFRP coupons was about 12%. Observed differences are not explained, but possible reason for the poorer performance of CFRP exposed to freeze-thaw cycles may be due to a mismatch in coefficient of thermal expansion (CTE) between the fiber and resin. CTE of carbon fibers has low negative value in the axial direction and high positive value in the radial direction, while resins have positive CTE. As a result of differential deformation during thermal stresses, CTE induced defects like cracking can lead to premature failure.

When EB FRP is employed in harsh environmental conditions in infrastructure, carbon fibers are utilized almost exclusively. Carbon fibers are inert to all environments typically experienced by civil infrastructure. They also offer superior mechanical properties and high resistance to creep-rupture. Carbon fibers possess better resistance to chemical attack than glass and aramid fiber. However, degradation of the outer layer of the fiber, which involves ion exchange reaction between the fiber and metal ions in the acid, results in the degradation of the interphase [82].

Many studies that were performed to assess the longevity of wet-layup carbon fiber reinforced polymer (CFRP) composites confirmed their excellent durability properties [84,96]. These studies agree that deterioration of composite’s performance under accelerated conditioning in a variety of environmental conditions (alkaline solution, fresh water, acidic, seawater, UV radiation) is negligible. Deterioration in the composite properties is attributed to degradation of the matrix and fiber–matrix interface rather than the fibers. However, the mechanical properties of CFRP composites may be compromised at elevated temperatures [97,98] due to the matrix “softening” effect (particularly when their *T*_g_ is exceeded). Exposure to freeze-thaw cycling can result in reduced mechanical properties as a result of thermal incompatibility of constituent materials, as explained above [81,99]. Effect of salts and acids solutions has been reported by many authors [82,100,101]. As a conclusion, this type of exposure results in degradation of matrix-dominated properties, while degradation of fiber-controlled properties (tensile strength and modulus) is negligible.

### 3.3. EB FRP Bond to Concrete

**Figure 11:**
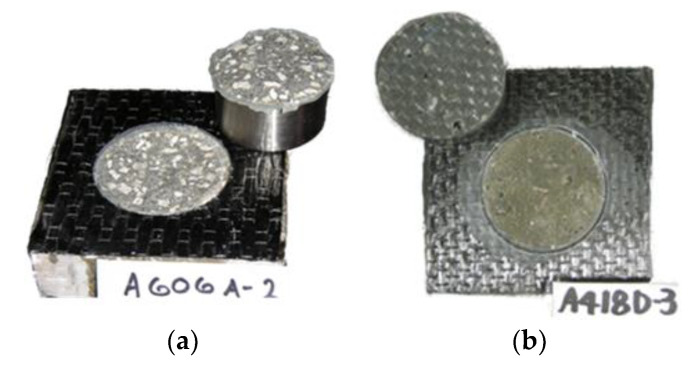
A typical shift in EB FRP/concrete pull-of bond test failure mode following accelerated conditioning in moisture: (**a**) cohesive failure mode in “dry” conditions and (**b**) adhesive failure mode after accelerated conditioning by water immersion.

Though EB CFRP composites show excellent durability under accelerated conditioning, the same cannot be said for the EB FRP-concrete adhesively bonded joints. Accelerated conditioning studies on EB FRP bonded to concrete have revealed varying levels of bond deterioration depending on the conditioning environment, conditioning time, and stress state (tensile vs. shear stress). Multiple researchers evaluated the durability of bond between EB FRP and concrete under moisture [12,102], dry heat [103], freeze-thaw cycles [104], alkaline environment [96], salt and moisture [105], wet/dry cycles [106], UV radiation [107], etc. Tatar and Hamilton [13] compiled a database of over 600 data points on bond strength deterioration from 25 studies. The dataset considered varying conditioning times, exposure conditions, composite manufacturers, adhesives, bond test methods, etc. The average loss in bond properties for the entire dataset was 15% with a standard deviation of 24%. The variation in data is quite significant as different levels of bond degradation were observed depending on the test variables, particularly exposure condition. Nonetheless, the data clearly indicated that accelerated conditioning can significantly deteriorate the EB FRP/concrete bond. Research, for the most part, agrees that moisture exposure is the most detrimental to the bond properties. The loss in bond strength due to moisture has also been linked to a change in failure mode from “cohesive” (failure within the concrete substrate) to interfacial separation between adhesive and substrate (also known as “adhesive” failure mode) indicating loss of adhesion (Figure 11) [108,109].

**Figure 12:**
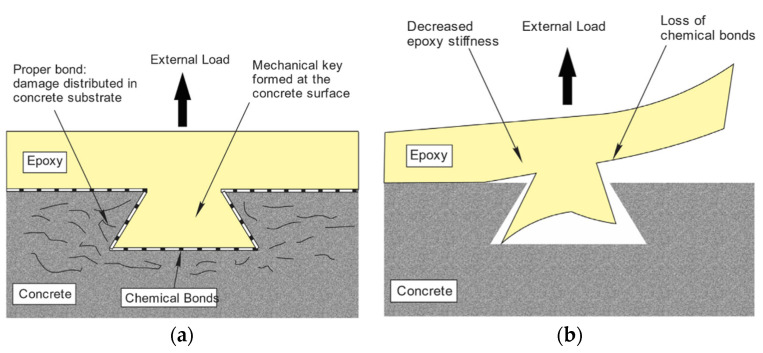
(**a**,**b**) Possible degradation mechanism of bonded joints (reprinted from [66] by permission from Elsevier).

The bond between epoxy and concrete is formed through chemical bonding and mechanical interlock. The primary chemical interaction between the adherents was found to be hydrogen bonding [110,111], which is orders of magnitude weaker than covalent bonding [112]. The flow of low-viscosity epoxy through the open pores, crevices, and irregularities on the roughened concrete surface forms the mechanical interlock. Following curing, the resin creates strong shear keys with the substrate that facilitate the transfer of stress between the EB FRP composite and concrete (Figure 12). It has been proposed that reduction of epoxy stiffness due to plasticization (or ambient temperature exceeding the *T*_g_ of epoxy) loosens the mechanical interlock which, combined with hydrogen bond breakage due to interactions by water molecules, leads to loss of adhesion and reduction in bond strength after prolonged exposure to moisture [50,66,113]. Relatively recent research also indicated that epoxy-concrete interfaces are susceptible to an increased rate of stress-corrosion cracking under moisture conditioning [114].

**Figure 13:**
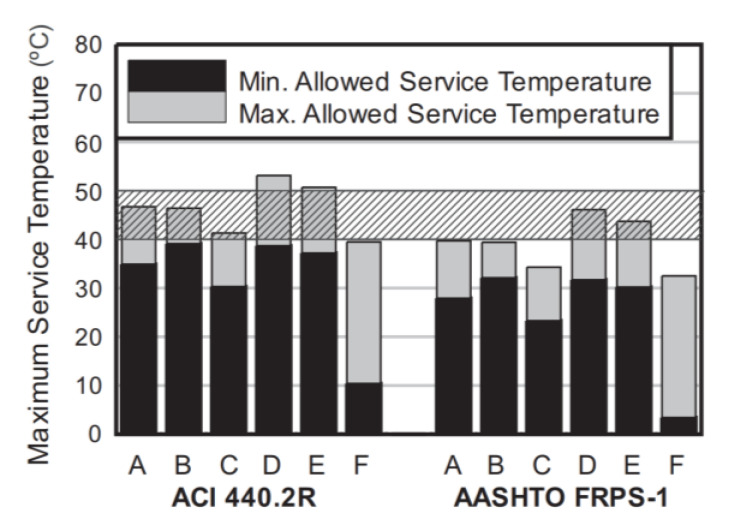
Maximum and minimum allowed service temperatures for six epoxy adhesives (A through F) calculated from glass transition temperature (*T*_g_) measurements based on ACI 440.2R and AASHTO FRPS-1 design guidelines; shaded region represents the typical maximum design temperature range per AASHTO 2017 (reprinted from [66] by permission from Elsevier).

Another threat from water exposure lies in its effect on the *T*_g_ of epoxy. As previously discussed, plasticization leads to a reduction in *T*_g_, which increases the possibility of ambient temperature exceeding the *T*_g_. Because of this, many design guidelines specify a minimum *T*_g_ for a resin. For instance, ACI 440.2R requires the *T*_g_ to be at least 15 °C higher than the maximum design temperature, while AASHTO FRPS-1 is a bit more conservative and requires the *T*_g_ to be at least 22 °C higher than the maximum design temperature. Blackburn et al. [66] measured the changes in *T*_g_ following hygrothermal conditioning for six typically used epoxy adhesives in FRP composites. The experimental data was used to compute maximum and minimum allowed service temperatures per ACI 440.2R and AASHTO FRPS-1 based on the maximum and minimum measured *T*_g_ in the accelerated conditioning experiments, respectively (Figure 13). The data indicated that none of the adhesives were suitable for applications within the typical design temperature range for the U.S. bridges, per ACI 440.2R and AASHTO FRPS-1.

**Figure 14:**
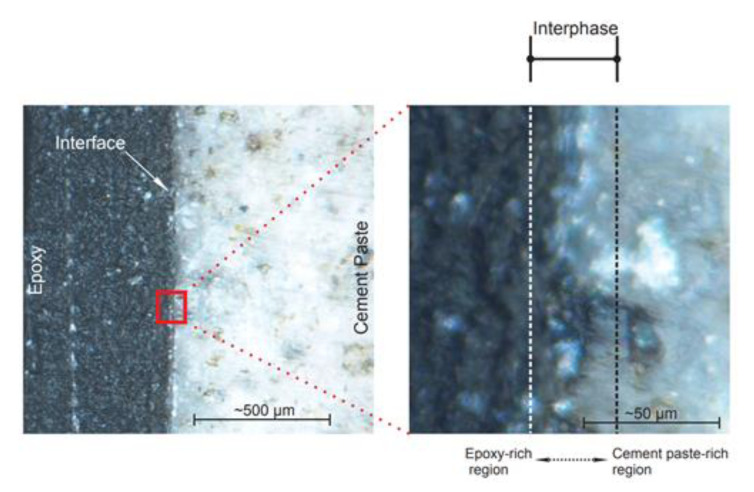
Stereo microscope photographs of epoxy-cement paste interfacial region at different length scales (reprinted from [115] by permission from Elsevier).

**Figure 15:**
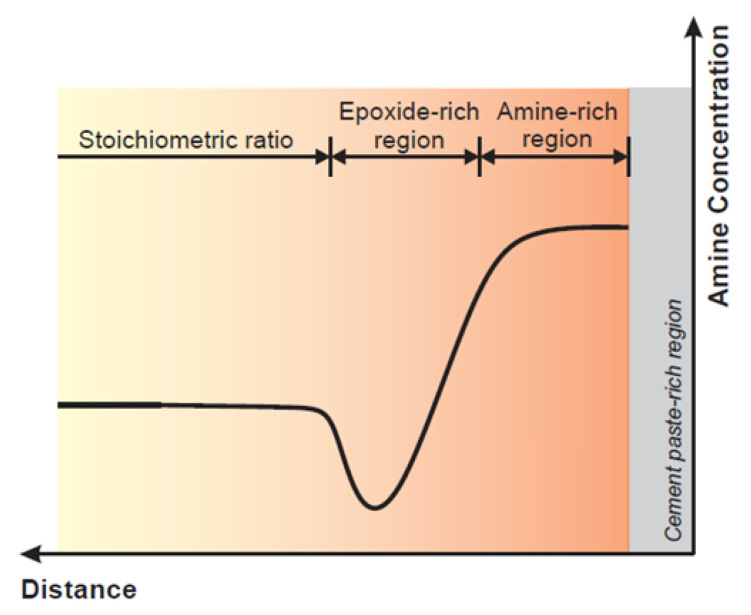
Supposed structure of epoxy-rich region within interphase (reprinted from [115] by permission from Elsevier).

The actual mechanism of bond deterioration might be even more complex than previously discussed. The plane of contact between the epoxy surface and the surface of the cement paste/concrete substrate is referred to as interface. From a macroscale point of view, the interface divides the two adherents. Although interface may be apparent at the larger length scales, recent research [34,115] showed evidence that a transition region, termed interphase, exists between bulk epoxy and bulk cement paste/concrete substrate (Figure 14). The presence of this region is caused by permeation of low-viscosity epoxy into the porous network of a cementitious material substrate, as well as the preferential reaction between amine-based hardener and cement hydrates [115]. This preferential reaction is deemed to cause a shortage of amines adjacent to the interface (as shown in Figure 15). As a result, it is thought that epoxy adjacent to the plane of contact between epoxy and substrate is characterized with lower cross-linking density than the bulk epoxy. This epoxide-rich region (lower degree of cure) is hypothesized to be adjacent to the amine-rich region as shown in Figure 15. The presence of epoxide-rich region could render the interface more vulnerable to environmental degradation [115].

As can be gleaned from the previous discussion, deterioration of EB FRP/concrete bond region is a complex phenomenon that is not well understood. As such, EB FRP/concrete bond service life estimation based on the accelerated conditioning data is a daunting task. Accelerated conditioning procedures are not directly related to real-world environmental conditioning and can, thus, result in either underestimation of durability, or overly conservative estimation of materials’ durability properties. To correlate laboratory accelerated conditioning data to realistic deterioration in the field conditions, one needs to understand the relationships between the service environment, deterioration mechanisms, rate of reactions, and property change—such relationships are currently elusive due to the complexity of possible degradation mechanisms and very few long-term durability data from the field [41,47,48,116,117].

Some researchers attempted utilizing Arrhenius law to extrapolate FRP-concrete bond long-term durability from the short-term accelerated conditioning data. It should be noted that such approach is fundamentally inappropriate because Arrhenius law applies only to an elementary singular process chemical reaction or a multiprocess reaction if the global rate of reaction equals the product of elementary process reaction rates [118]. Since the deterioration of the bond region is governed by multiple competing processes, it is not prudent to assume that underlying assumptions behind Arrhenius law are satisfied. Hence, there is a need to develop new and improved multi-reaction deterioration models to allow for correlation between accelerated conditioning and field conditioning and enable accurate service life estimation for EB FRP composite systems in concrete structures.

### 3.4. Materials Development

The inherent sensitivity of the strengthening system to moisture, in particular EB FRP/concrete adhesive bond, remains a significant problem. Relatively recent developments in materials have led to adhesives with improved mechanical and durability properties. Addition of nanoparticles, such as nanoclays [119], carbon nanotubes [120,121], graphene [122], nanosilica [123,124], and core-shell rubber nanoparticles [125,126], to name a few, to the base resin can result in adhesives and FRP composites with enhanced properties.

**Figure 16:**
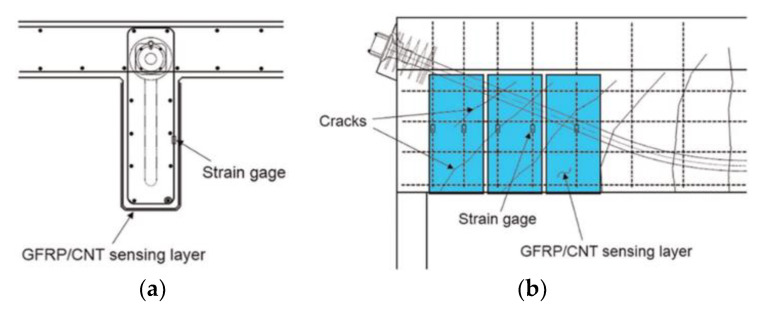
The principle of distributed sensing shown on a prestressed concrete beam section: (**a**) cross section and (**b**) elevation view. GFRP: glass fiber–reinforced polymers; CNT: carbon nanotube (reproduced from [129] with permission from SAGE Publications).

For instance Aboubakr and Kandil [119] demonstrated that addition of nanoclays to the base epoxy can significantly improve the performance of FRP-steel bonded joints under sustained loading by reducing the creep compliance of the adhesive. A recent study by [125] showed that addition of nanoparticles, particularly core-shell rubber nanoparticles, to the epoxy adhesive can enhance the durability of EB FRP/concrete bond under hygrothermal conditions. Enhancements in bond strength, bond durability, and an increase in *T*_g_ for ambient cured nanosilica-epoxy nanocomposites were reported as well [123,124]. Ghosh et al. [122] achieved significant improvements in GFRP creep-rupture resistance with the addition of graphene to the composite matrix. Besides mechanical and durability property enhancements, conductive nanoparticles such as carbon nanotubes and carbon nanofibers can be utilized to add self-sensing capabilities to the EB FRP composite [127,128] or the adhesive layer [129] allowing for distributed sensing over a large area and detection of damage (Figure 16).

**Figure 17:**
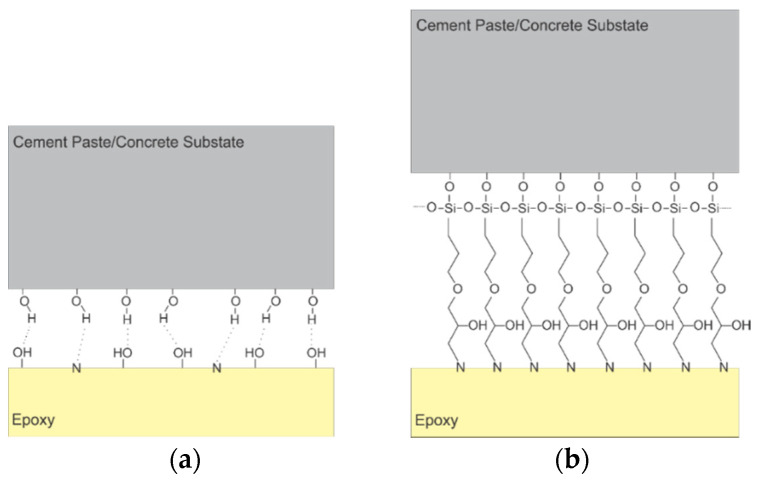
Proposed interactions at the interface between epoxy and cement paste/concrete: (**a**) hydrogen bonding and (**b**) covalent bonding via 3-glycidoxypropyltrimethoxysilane (GPTMS) coupling agent.

Improvements in the epoxy/concrete bond durability have also been reported with concrete surface pre-treatment (before adhesive application) with epoxy-functional silane coupling agents [65,114,126,130]. Per Stewart et al. [131], epoxy-functional silanes can form covalent bonds with the concrete substrate which allows for stronger adhesive interaction between epoxy and the cementitious substrate (Figure 17). However, the effect of cohesive mechanical properties and durability of the silane layer on the bond durability are still not well understood and warrant further research [130].

## 4. Design Guidelines

### 4.1. Overview of Design Guidance

To date, there are no prescriptive design codes that specify the requirement for the design of EB FRP in concrete structures which is thought to be one of the one of the barriers to a more widespread adoption of EB FRP systems. There are, however, various design guidelines that were developed in different countries. Some of the notable documents providing design guidance are the following:

- United States: American Concrete Institute (ACI) 440.2R-08: “Guide for the Design and Construction of Externally Bonded FRP Systems for Strengthening Concrete Structures”; American Association of State and Highway Transportation Officials (AASHTO) FRPS-1, [132]: “Guide Specifications for Design of Bonded FRP Systems for Repair and Strengthening of Concrete Bridge Elements”.
- Canada: Intelligent Sensing for Innovative Structures (ISIS) [133] Design Manual 4, FRP Rehabilitation of Reinforced Concrete Structures.
- Japan: Japan Society of Civil Engineers (JSCE) [134]. “Recommendations for Upgrading of Concrete Structures with use of Continuous Fiber Sheets”.
- United Kingdom: United Kingdom Concrete Society Technical Report 55 (TR55) [135]. “Design Guidance for Strengthening Concrete Structures Using Fibre Composite Materials”.
- Italy: Italian National Research Council Technical Document 200 (CNR-DT200) [136]. “Guide for the Design and Construction of Externally Bonded FRP Systems”.

Given that the focus of this paper is mainly on the environmental durability of EB FRP in bond-critical applications, pertinent information regarding environmental reduction factors and maximum usable strain in EB FRP for flexure design is summarized in Table 1. It can be noted that only ACI 440.2R and CNR-DT 200 explicitly specify environmental reduction factors for different fibers and environmental conditions. These factors are used to reduce the EB FRP design rupture strain to account for the effects of FRP composites deterioration. The source of the proposed environmental reduction factors is not clear. Anecdotal evidence suggests they were selected to reflect the comparative differences in durability between different fibers, rather than being calibrated by a substantial experimental database. Neither design guideline suggests an explicit consideration of EB FRP/concrete bond durability.

ISIS and TR55, while not providing explicit environmental reduction factors, differentiate between the performance of different types of EB FRP based on the fiber material and manufacturing procedure by implementing the material safety factors. It is believed that these factors account for variations in durability between different types of EB FRP. In that regard, ISIS is a bit more explicit in that it provides a different set of material safety factors for buildings and bridges. The maximum usable strain in EB FRP in flexural design is limited to 0.006 and 0.008 in ISIS and TR55, respectively.

AASHTO FRPS-1 guide specification does not propose explicit environmental reduction factors. However, it is specified that EB FRP composite shall retain at least 85% of glass transition temperature (*T*_g_) determined per ASTM E1640, and characteristic strain determined per ASTM D3039 [137] following accelerated conditioning in four specified environments: (i) water, (ii) alternating ultraviolet light and condensation humidity, (iii) alkali, and (iv) freeze-thaw. AASHTO FRPS-1 also suggests that EB FRP composites should achieve a minimum strain of 1% to qualify for bridge applications. For flexural EB FRP strengthening design, AASHTO FRPS-1 recommends a maximum usable strain of 0.005 which is based on the available experimental evidence. While EB FRP/concrete bond durability is not explicitly accounted for by the design factors, AASTO FRPS-1 has a requirement that EB FRP/concrete bond shall retain a minimum strength of 200 psi or $0.171\sqrt{{f^{\prime }}_{c}}$ (${f^{\prime }}_{c}$ is specified compressive strength of concrete in MPa), whichever is greater, following conditioning in the specified accelerated conditioning protocols. The test method used to determine the bond strength is to be specified by the licensed design professional.

Mechanical anchorage of EB FRP can increase the effective usable strain in the composites in bond-critical applications [138,139]. Effective use of anchoring devices can lead to a change in failure mode from debonding to composite rupture [140]. Many design guidelines recognize anchorage as an effective tool for improving the performance of bond-critical EB FRP; however, specific design guidance of anchorage systems is lacking at this time.

### 4.2. Standard Test Method for EB FRP/Concrete Bond Durability

**Figure 18:**
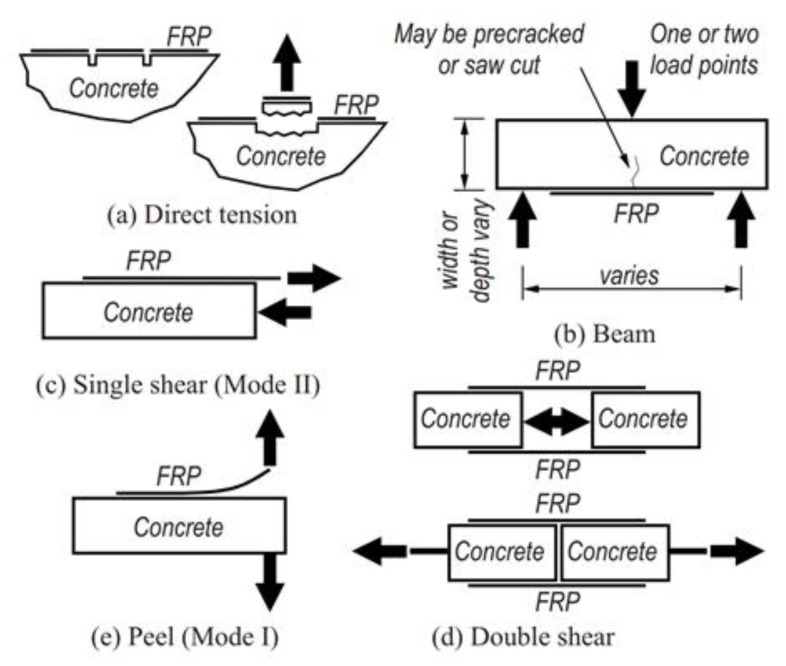
(**a**–**e**) Different types of bond test methods in the literature (reproduced from [143] by permission from American Society of Civil Engineers).

EB FRP/concrete bond durability is not explicitly addressed by the existing design guidelines even though it may be the governing factor in long-term durability of EB FRP systems [13]. There have been multiple efforts to develop a standard test method and an accelerated conditioning protocol for evaluation of EB FRP-concrete bond durability [102,142,143]. Some of the common test methods utilized in the literature are schematically shown in Figure 18. Pull-off style tests (Figure 18a,c–e) are popular as they allow to simulate specific loading conditions (e.g., pure tension vs. pure shear) but can be difficult to execute in a laboratory setting due to the misalignment problems. On the other hand, beam style tests (Figure 18b) are simpler to execute and can mimic realistic stress states experienced by EB FRP in flexural strengthening applications.

**Figure 19:**
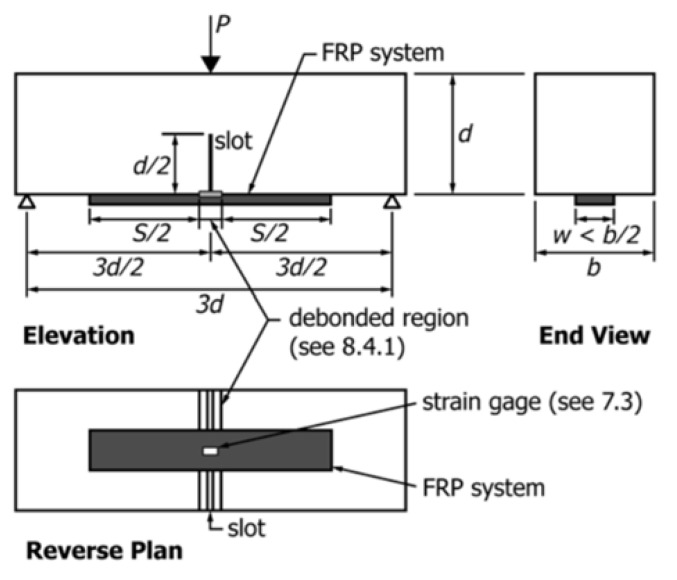
Typical test configuration (reproduced, with permission from [114], copyright ASTM International, 100 Barr Harbor Drive, West Conshohocken, PA 19428).

A particularly popular test is a notched beam three-point bending test [143]. The test utilizes a common concrete prism specimen used in standard Modulus of Rupture (MOR) test (ASTM C78, [144]) with a notch at the midspan (Figure 19). Notch is introduced to simulate cracked concrete while also allowing for a predetermined debonding path. This test was standardized (ASTM D7958) [145] and implemented in the recent American Concrete Institute durability evaluation guide—ACI 440.9R [25]. This document is likely the only of its kind that provides specific guidance for durability evaluation of EB FRP/concrete bond durability. ACI 440.9R sets the framework for EB FRP/concrete bond durability evaluation, but it is still not defined how the durability test data can be used in the design of EB FRP.

Informed by the research findings, ACI 440.9R also specifies a standard accelerated conditioning protocol for notched beams with EB FRP consisting of 3000-hour conditioning by water immersion at 50 ± 3 °C. The durability of the bond is quantified via bond strength retention ($R_{b}$), as follows:

(1) $$R_{b}=\frac{P_{ACP}}{P_{SLC}}$$

where $P_{SLC}$ is the average strength of specimens kept in standard laboratory conditions, and $P_{ACP}$ is the average strength of specimens subjected to the accelerated conditioning protocol.

## 5. Concluding Remarks

As the existing infrastructure is aging worldwide, there is an immense need to develop and implement novel strengthening methods to prolong the service life thereof. EB FRP composites represent one of the most economical solutions. However, the long-term durability of EB FRP in harsh environments may limit the efficacy of these systems. As it was demonstrated in this article, there are multiple competing degradation mechanisms in the epoxy resins, fibers, fiber–epoxy interface, and epoxy–concrete interface that can affect the performance of EB FRP. Recent research on nanomodified resins demonstrates potential to develop improved materials capable of withstanding harsh environmental and loading conditions.

While design guidelines consider the durability of EB FRP an important factor, there is quite a lot of variability in how the durability concerns are addressed. Some design guidelines provide explicit environmental reduction factors, while in others either material’s qualification criteria are proposed, or durability is accounted for through material safety factors. Probably the most inconsistent between the guidelines is the maximum usable strain in FRP for flexural EB FRP design—some guidelines propose specific strain limits while others offer debonding strain equations that compute maximum usable strain in EB FRP based on the substrate and composite properties.

## 6. Future Perspectives and Recommendations

Based on the review of relevant research, the authors offer the following insights:

1. The complexity of the involved deterioration mechanisms limits our ability to mechanistically model the EB FRP deterioration under realistic environmental exposures and develop accurate service life prediction models from the short-term accelerated conditioning test data. This is one of the top research priorities. Progress in EB FRP service life estimation will lead to refined durability design guidelines that will allow for economical yet safe EB FRP strengthening.
2. Interestingly, even though the durability of the bond between EB FRP and concrete is a critical factor, explicit treatment of EB FRP/concrete bond durability is not suggested by any of the available design guidelines. It is, thus, imperative that these concerns be addressed either through qualification testing requirements, bond durability design factors, or both.
3. Advancements in materials for EB FRP applications are necessary to meet the performance requirements imposed on EB FRP strengthening systems in harsh environments. Future progress in composites for construction likely lies at an intersection between classical structural engineering and materials science. With the emerging materials and technologies, an interdisciplinary approach to addressing the problems in the next-generation infrastructure will be paramount.

## Figures and Tables

**Table 1:** Summary of flexural design guidance for EB FRP.

Design Guideline	Environmental Reduction Factors			Maximum Usable Strain in FRP for Flexure ^
ACI 440.2R	*Exposure condition*	*Fiber*	$C_{E}\;$ *^*	$\varepsilon_{u}=\min\left\{\varepsilon_{fd}=0.41\sqrt{\frac{{f^{\prime }}_{c}}{nE_{f}t_{f}}},0.9C_{E}\varepsilon_{fu}\right\}$
	Interior Exposure	Carbon	0.95	
		Glass	0.75	
		Aramid	0.85	
	Exterior Exposure (bridges, piers and unclosed parking garages)	Carbon	0.85	
		Glass	0.65	
		Aramid	0.75	
	Aggressive environment (chemical plants and wastewater treatment plants)	Carbon	0.85	
		Glass	0.50	
		Aramid	0.70	
AASHTO FRPS-1	No environmental reduction factors proposed. Specified that composite shall retain at least 85% of glass transition temperature (Tg) determined per ASTM E1640 and characteristic strain determined per ASTM D3039 following accelerated conditioning in four specified environments: (i) water, (ii) alternating ultraviolet light and condensation humidity, (iii) alkali, and (iv) freeze-thaw.			$\varepsilon_{u}=0.005$
ISIS	No explicit environmental reduction factors proposed. Material safety factors that account for fiber type and composite manufacturing procedure are explicitly specified. The material safety factors make a differentiation between buildings and bridges to account for the environmental effects.			$\varepsilon_{u}=0.006$
JSCE	Environmental reduction factors not explicitly specified. Suggested to use a protective layer (coating, mortar, or concrete) in outdoor applications, unless it can be demonstrated by suitable numerical simulation and accelerated conditioning tests that protection is not necessary. The designer is referred to JSCE “Standard Specifications for Design and Construction of Concrete Structures (Design)” for selection of material safety factors.			$\varepsilon_{u}=\min\left\{\varepsilon_{fd}=\sqrt{\frac{G_{f}}{nE_{f}t_{f}}},\varepsilon_{fu}\right\}$
TR55	Environmental reduction factors not explicitly proposed. Material safety factors depending on fiber type (carbon, aramid, and glass) and manufacturing procedure are specified.			$\varepsilon_{u}=\min\left\{\varepsilon_{fu},\;0.008\right\}$
CNR-DT 200	Same as ACI 440.2R; however, it is stated that: “Designer shall use these values when more information on test evidence for the material in use and expected environmental condition are missing.”			$\varepsilon_{u}=\min\left\{\varepsilon_{fd}=0.373\sqrt{k_{b}\frac{\sqrt{{f^{\prime }}_{c}f_{ct}}}{nE_{f}t_{f}}},C_{E}\varepsilon_{fu}\right\}$ (typical design case)

^ Variables and units: $C_{E}$, environmental reduction factor; $\varepsilon_{fu}$, design rupture strain of EB FRP; $\varepsilon_{fd}$, debonding strain; ${f^{\prime }}_{c}$, specified compressive strength of concrete (MPa); $f_{ct}$, tensile strength of concrete (MPa); n, number of EB FRP plies; $E_{f}$, modulus of elasticity of EB FRP (MPa); $t_{f}$, nominal thickness of a single EB FRP ply (mm); $G_{f}$, interfacial fracture energy between EB FRP and concrete determined based on JSCE-E 543-2000 [141] standard test method, or taken as 0.5 N/mm in absence of experimental data; $k_{b}$ is geometrical correction factor computed as $k_{b}=\sqrt{\left(2-b_{f}/b\right)/\left(1+b_{f}/b\right)}\geq 1.0$ for $b_{f}/b>0.25$ (if $b_{f}/b<0.25$ then $k_{b}=1.18$) where $b_{f}$ is the width of EB FRP sheet, and b is the width of concrete section.
